# Long-Term External Counterpulsation Reduces Beat-to-Beat Blood Pressure Variability Without Changing Arterial Blood Pressure in Ischemic Stroke: A Retrospective Case-Control Study

**DOI:** 10.3390/bioengineering13050520

**Published:** 2026-04-29

**Authors:** Lixia Zhu, Xinyi Chen, Xiaoling Li, Thomas W. Leung, Lawrence Ka Sing Wong, Jack Jiaqi Zhang, Yiao Liu, Bin Luo, Jianhang Du, Yiliang Li, Li Xiong

**Affiliations:** 1 Pharmaceutical Department, The Eighth Affiliated Hospital, Sun Yat-sen University, Shenzhen 518033, China; alicesuly@163.com; 2 Division of Neurological Rehabilitation, Department of Geriatrics, The Eighth Affiliated Hospital, Sun Yat-sen University, Shenzhen 518033, China; chenxy967@mail2.sysu.edu.cn; 3 Community Health Service Management Center, The Eighth Affiliated Hospital, Sun Yat-sen University, Shenzhen 518033, China; lixling27@mail.sysu.edu.cn; 4 Division of Neurology, Department of Medicine & Therapeutics, The Chinese University of Hong Kong, Hong Kong SAR 999077, China; drtleung@cuhk.edu.hk (T.W.L.); ks-wong@cuhk.edu.hk (L.K.S.W.); 5 Department of Rehabilitation Sciences, The Hong Kong Polytechnic University, Hong Kong SAR 999077, China; jack-jiaqi.zhang@polyu.edu.hk; 6 Division of cerebrovascular diseases, Department of Neurosurgery, The Eighth Affiliated Hospital, Sun Yat-sen University, Shenzhen 518033, China; liuyao26@mail.sysu.edu.cn (Y.L.); luob9@mail.sysu.edu.cn (B.L.); 7 Medical Research Center, The Eighth Affiliated Hospital, Sun Yat-sen University, Shenzhen 518033, China; 8 Department of Rehabilitation, Renmin Hospital of Wuhan University, Wuhan 430060, China; 9 Clinical Research Department, The Eighth Affiliated Hospital, Sun Yat-sen University, Shenzhen 518033, China

**Keywords:** blood pressure, blood pressure variability, external counterpulsation, ischemic stroke

## Abstract

**Background and purpose**: Short-term external counterpulsation (ECP) noninvasively augments cerebral blood flow by elevating blood pressure in ischemic stroke. The current retrospective case–control study examined the effect of long-term ECP treatment on blood pressure and beat-to-beat blood pressure variability (BPV) in patients with recent ischemic stroke. **Method**: The ECP group included data from 20 recent ischemic stroke patients who received five daily 1 h sessions each week for seven weeks, for a total of 35 sessions of ECP treatment from our ECP registry. An equivalent comparative control group without ECP treatment was composed from the same pool of patients and matched with cases by sex and age. Beat-to-beat heart rate and blood pressure were monitored before and after the long-term intervention. Power spectral analysis calculated the beat-to-beat BPV oscillations at very low frequency (VLF; <0.04 Hz), low frequency (LF; 0.04–0.15 Hz), high frequency (HF; 0.15–0.40 Hz), and the total power spectral density (TP; <0.40 Hz) and LF/HF ratio. **Result**: There was a significant reduction in systolic blood pressure (SBP) after the intervention compared with that before intervention in both groups (*p* < 0.05), but only the ECP group displayed a statistically significant reduction in diastolic blood pressure (DBP) (*p* = 0.023). The changes in SBP and DBP (delta SBP and delta DBP) from pre-intervention to completion showed no significant differences between the two groups (all *p* > 0.05). The ECP group exhibited a more pronounced and significant decrease in each spectral component of BPV after the intervention than at pre-intervention, with a substantial decrease in systolic BPV at TP (*p* = 0.048) and in the LF/HF ratios (*p* = 0.021 in diastolic BPV and *p* = 0.004 in systolic BPV, respectively) compared to the control group. **Conclusions**: A standard 35-session ECP treatment decreases beat-to-beat BPV but does not change SBP and DBP in patients with recent ischemic stroke. This implies that long-term ECP treatment may enhance autonomic regulation to benefit post-stroke clinical outcomes.

## 1. Introduction

Hypertension is the primary modifiable risk factor for ischemic stroke. The recommendations for blood pressure (BP) management in acute and chronic phases of ischemic stroke vary across the 2025 American Heart Association (AHA) [1], 2024 European Society of Cardiology (ESC) [2], 2023 European Society of Hypertension (ESH) [3], and 2025 Japanese Society of Hypertension (JSH) [4] guidelines. In acute ischemic stroke without reperfusion therapy, all four guidelines discourage routine BP lowering unless systolic BP (SBP) is ≥220 mmHg or diastolic BP ≥ 120 (110) mmHg, and then recommend only modest reductions of about 15% within 24 h. For patients receiving intravenous thrombolysis or mechanical thrombectomy, the guidelines converge on pre-treatment BP < 185/110 mmHg and maintenance < 180/105 mmHg during the first 24 h, with the JSH specifying micro-infusion calcium channel blockers as preferred agents. In chronic ischemic stroke, the AHA, ESH, and JSH generally endorse BP < 130/80 mmHg, whereas the ESC prioritizes an SBP range of 120–129 mmHg. Despite regional nuances, the guidelines converge on conservative management in acute ischemic stroke, and intensive long-term BP control as the global benchmark for secondary cerebrovascular prevention. However, a growing body of research suggests that good control of BP alone cannot completely prevent stroke. Blood pressure variability (BPV), rather than absolute BP levels, has received considerably less attention in acute stroke research, and current guidelines do not consider BPV in their recommendations. Observational studies have reported that BPV correlates more strongly with acute stroke prognosis than mean BP levels [5,6] and that the benefits of some antihypertensive drugs in the prevention of stroke may partly result from reduced variability in BP [7,8,9]. These findings may represent an important target for developing and evaluating future BP management interventions.

Depending on the measurement method and time interval, BPV can be classified into three types: long-term BPV (measured over exponential weeks, months and years), short-term BPV (ambulatory BP monitoring over 24 h every 15–20 min) and very short-term BPV (variability in BP from one beat to the next, beat-to-beat BPV). Notably, both long-term and short-term BPV require a prolonged period of assessment, good patient compliance, and follow-up visits. As such, they are of limited use in acute assessment. Beat-to-beat BPV enables noninvasive assessment at a single visit to record the physiological and pathological information hidden in the time series of blood pressure. It is more convenient and easier to implement in clinical practice. Most studies have demonstrated that beat-to-beat BPV, with a similar physiological profile to home day-to-day BPV [10], has diagnostic and predictive value in patients with a transient ischemic attack (TIA) or acute ischemic stroke [11,12,13]. Therefore, beat-to-beat BPV provides a meaningful therapeutic target in stroke management and prevention.

External counterpulsation (ECP) is a mature and noninvasive method of treating ischemic cardiovascular disease [14,15]. ECP triggers the inflation of lower limb airbags during the diastolic phase and deflation during the systolic phase through electrocardiogram monitoring. This enhances diastolic blood flow and reduces cardiac systolic resistance, thereby improving the perfusion of the heart and the brain, and may optimize vascular function in the long term [16]. Our previous study showed that ECP is safe and may help the recovery of ischemic stroke patients with cerebral large artery occlusions [17]. Short-term ECP intervention could reduce beat-to-beat BPV while increasing BP and cerebral blood flow velocity in patients with ischemic stroke [18,19]. A three-minute ECP treatment period was applied in these studies, instead of the standard treatment regimen, which consists of five daily 1 h sessions each week for seven weeks, for a total of 35 sessions. The long-term effects of ECP treatment on beat-to-beat BPV and BP in ischemic stroke patients are still unclear. In this study, we aimed to evaluate the impact of a standard ECP treatment course on beat-to-beat BPV in patients who have experienced an acute ischemic stroke. Previous reports have used data from the EECP registry to investigate various effects of EECP [15,20]. We also identified retrospectively the significant predictors of good functional outcomes for ECP-treat ischemic stroke from our ECP registry which includes stroke patients only [21]. The purpose of this study was to retrospectively discover the significant effects of long-term standard treatment regimen on beat-to-beat BPV in ischemic stroke patients from our ECP registry.

## 2. Methods

### 2.1. Patients

We enrolled consecutive patients with recent ischemic stroke in the anterior circulation or posterior circulation territories, who were hospitalized in the Acute Stroke Unit at Prince of Wales Hospital. Diagnosis of stroke was made based on the definition of the World Health Organization, and ischemia was confirmed by CT or MRI. The main exclusion criteria included: (1) cardioembolic stroke such as atrial fibrillation and rheumatic heart disease; (2) evidence of hemorrhage on brain CT; (3) history of intracranial hemorrhage; (4) evidence of arteriovenous malformation, arteriovenous fistula or aneurysm, (5) concurrent major morbidity, including renal failure, severe dementia, impaired consciousness, psychosis or active malignancy; (6) sustained severe hypertension (systolic > 180 mm Hg and/or diastolic > 100 mm Hg), sepsis, hypoxia, or hemodynamic instability including circulatory shock and/or advanced heart failure; (7) receipt of reperfusion therapy (intravenous thrombolysis, mechanical thrombectomy or endovascular stenting). Patients who agreed to receive ECP treatment as an additional therapy to their routine medical treatment were given ECP therapy, and their data were entered into our registry. Among those stroke patients recruited from November 2017 to December 2019, we included 20 recent ischemic stroke patients who received five daily 1 h sessions each week for seven weeks, for a total of 35 sessions of ECP treatment as the ECP group, using an Enhanced External Counterpulsation system (model number MC2, Vamed Medical Instrument Company, Foshan, China, cuff inflation pressure between 150 and 225 mm Hg). The interval from stroke onset to recruitment was not >14 days. We also recruited 20 hospitalized ischemic stroke patients during the same period, who fulfilled the same inclusion and exclusion criteria as the ECP group but refused ECP and received only medical treatment as the control group.

### 2.2. Measurement of BP and Beat-to-Beat BPV

Continuous monitoring of heart rate, BP, and respiration was recorded using a Task Force Monitor 3040i (CNSystems Medizintechnik AG, Graz, Austria). All patients were asked to refrain from caffeine ingestion on the day of the investigations and to take only a light breakfast. All investigations were performed between 9:00 a.m. and 11:00 a.m. in a warm, quiet room. To minimize deviations caused by individual breathing patterns, we recorded heart rate, BP, and respiratory rate within five minutes of a resting period. The respiratory rate was set to fifteen breaths per minute after an initial ten-minute adjustment period. BP was measured through finger cuffs on the index and middle fingers of the left hand with appropriate cuff sizes (small, medium, or large). The BP and respiratory signals were first edited automatically, then sampled digitally and transferred from the Task Force Monitor 3040i system to a computer for analysis of the beat-to-beat BPV using power spectral methods.

For spectral analysis, histograms of the SBP and diastolic BP (DBP) for sinus beats were computed and digitized at 10 samples per second. Autoregressive modeling was used to construct frequency domain spectrograms of the BPV [22]. The BPV included oscillations at very low frequency (VLF; <0.04 Hz), low frequency (LF; 0.04–0.15 Hz), and high frequency (HF; 0.15–0.40 Hz); additionally, the total power spectral density (TP; <0.40 Hz) and LF/HF ratio were calculated [23]. Evidence shows that myogenic vascular function, the renin-angiotensin system (RAS), and endothelium-derived nitric oxide affect BPV at VLF [23,24], whereas the LF BPV reflects changes in sympathetic activity. In addition, the HF BPV is mainly influenced by changes in cardiac output, and the LF/HF ratio has been validated as a marker of sympathetic vascular activity and short-term autonomic regulation [23].

### 2.3. Data Analysis

Distributions of continuous variables were determined by the Kolmogorov–Smirnov test. In case of a normal distribution, mean values (±standard deviation) were calculated for continuous variables. The median and interquartile were presented for variables with a non-normal distribution, and frequencies were measured for categorical variables. Comparison between groups for variables with a normal distribution was calculated using the two-tailed Student’s *t*-test, or the Mann–Whitney U test for non-normally distributed variables. Group differences for categorical variables were examined using the χ^2^ test or Fisher’s exact test, as appropriate. In particular, Fisher’s exact test was applied in case of an expected count < 5. All statistical analyses were calculated using SPSS version 24.0 (SPSS Inc., Chicago, IL, USA). A two-tailed *p* value < 0.05 was considered statistically significant.

### 2.4. Ethical Approval

All participants provided written informed consent form prior to enrollment. The study was approved by the Institutional Ethics Committee (Joint CUHK-NTEC Clinical Research Ethics Committee, CREC Reference No.: 2016.489).

## 3. Results

### 3.1. Baseline Characteristics

The study retrospectively recruited a total of 40 recent ischemic stroke patients, with a mean age of 63.2 ± 9.2 years, including 87.5% males, and 12.5% females. Stroke patients had moderate neurological deficits with a mean admission NIHSS score of 6.6. The mean interval from stroke onset to examination was 7.9 days. The grouping method was based on the type of intervention, with 20 patients receiving only conventional medical treatment and 20 patients undergoing extra ECP treatment. There were no significant differences in mean age and gender between the two groups. Comparison of common risk factors and complications had similar proportions, displaying no statistical significance. The stroke infarct locations, subtypes and the use of medications for the patients in the two groups were also roughly similar. Detailed baseline characteristics of participants are displayed in Table 1.

### 3.2. Systolic and Diastolic Blood Pressure

Table 2 includes a comparison of SBP and DBP between the two groups at baseline and after the completion of the 35-session ECP treatment. At baseline and after the intervention, SBP and DBP were comparable between the two groups. The changes in SBP and DBP (delta SBP and delta DBP) from pre-intervention to intervention completion showed no significant differences between the two groups (all *p* > 0.05). In the comparison of SBP and DBP at the two measurement points within the groups, SBP decreased significantly in both groups: the ECP treatment group (*p* = 0.006) and the control group with medical treatment (*p* = 0.012); however, for DBP, a significant decrease was observed in the ECP group (*p* = 0.023), while the reduction in the control group did not reach statistical significance (*p* = 0.073).

### 3.3. Beat-to-Beat BPV

Diastolic BPV analysis (Table 3) identified that in the control group, LF, HF, and TP components were significantly lower, whereas VLF diminished only non-significantly and the LF/HF ratio significantly increased by 95.13% at the second measurement point after the intervention compared with pre-intervention. The patients in the ECP group showed a significant reduction in almost all spectral components, including VLF, LF, HF, and TP. Comparing the two groups, the change in reduction in the LF/HF ratio was significantly greater in the ECP group (decreased 13.33%, *p* = 0.021).

Systolic BPV analysis (Table 4) indicated only a significant decrease in HF in the medical group, while the ECP group demonstrated significant and steady declines in VLF, LF, HF, and TP between the two measurement points. The LF/HF ratio significantly increased by 71.82% at the second measurement point after the intervention compared with pre-intervention. Between-group comparison verified that the decreases in TP (*p* = 0.048) and LF/HF ratio (*p* = 0.004) were significantly greater in the ECP group compared to the control group.

## 4. Discussion

In this retrospective case–control study, we found that among the BPV frequency domain components (VLF/LF/HF/TP), the ECP group demonstrated a more extensive and more significant downward trend, and the LF/HF ratio in both SBP and DBP variability, which reflects sympathetic vascular activity, showed a significant decrease. This may indicate that a long-term 35-session ECP treatment contributes to the amelioration of autonomic nerve balance by decreasing sympathetic drive. Although it did not change arterial SBP and DBP values, which may be related to concurrent antihypertensive medications effect, an additional standard 7-week 35 sessions of ECP treatment could more effectively reduce beat-to-beat BPV compared with traditional medication treatment alone in patients with recent ischemic stroke.

For beat-to-beat BPV, early investigators implemented invasive intra-arterial recording and demonstrated that an increase in beat-to-beat BPV was directly related to the severity of target organ damage in hypertensive patients [25]. However, the monitoring methodology was invasive and could not be popularized in the clinic. In the late 1980s, with the emergence of Finapres technology, noninvasive beat-to-beat BP monitoring was utilized with high accuracy and precision via finger arterial pressure [26]. Some studies showed that beat-to-beat BPV could provide more prognostic information than other types of BPV. Dawson et al. compared casual and five min beat-to-beat BP recordings on outcome after acute ischemic stroke within 72 h of ictus. They observed that the odds ratio for a poor outcome was 1.38 (*p* = 0.014) for every 10 mm Hg increase in mean arterial pressure and 1.32 (*p* = 0.02) for every 1 mm Hg increase in mean arterial pressure variability, whereas casual BP measurements had no prognostic significance [27]. Webb et al. also compared different types of BP within six weeks of transient ischemic attack and non-disabling acute ischemic stroke onset in the Oxford Vascular Study including beat-to-beat (once a day for 5 min), day-to-day (continuous home monitoring for a week, three times a day), and ambulatory blood pressure monitoring. These outcomes revealed that beat-to-beat BPV predicted stroke and its recurrence, whereas ambulatory and day-by-day BPV lacked this effect [11]. Ren et al. found the predictive significance of beat-to-beat BPV within 24 h of acute ischemic stroke onset rather than 72 h or seven days. Compared with conventional clinical models including stroke risk factors and clinical characteristics, the addition of beat-to-beat BPV within 24 h of acute ischemic onset resulted in greater reclassification improvement. The nomogram indicated that the contribution of BPV parameters to the total final poor prognostic score was comparable to the NIHSS score at admission, which reflects the severity of the infarction [13]. Therefore, beat-to-beat BPV monitoring may be used as an early, rapid, efficient, and convenient monitoring method to provide important prognostic information for acute stroke patients. In this study, most parameters of SBP and DBP variability significantly increased in both groups within 14 days after acute ischemic stroke compared with the second measurement point after intervention completion. Our findings are consistent with those of previous studies and strongly suggest that beat-to-beat BPV increases following acute ischemic stroke. Conversely, our study mainly focused on the effect of ECP treatment on beat-to-beat BPV, which showed that ECP reduces sympathetic activity by decreasing the LF/HF ratio in recent ischemic stroke. Our pilot study showed that ECP is feasible for improving the clinical neurological deficit in ischemic stroke patients with large artery disease [17]. This implies that ECP enhances the prognosis of stroke patients by improving the autonomic regulation function.

BPV contributes to microvascular dysfunction, particularly in organs with low vascular resistance, such as the brain, kidneys, and eyes. It increases pulsatile pressure and reduces perfusion, causing structural and functional damage. The mechanisms underlying the elevation of beat-to-beat BPV are complicated, and the physiological basis of BPV relies on central and autonomic nerve stimuli, humoral stimuli and emotional factors such as stress, as well as the interaction between aortic compliance and systemic volume [28]. In recently diagnosed hypertensive subjects, inflammatory markers such as plasma high-sensitivity C-reactive protein (hsCRP) and soluble (s) E-selectin levels are related to awake SBP variability using the standard deviation of 24 h BP recordings. High SBP variability is likely associated with vascular inflammation in newly diagnosed hypertension, independent of SBP [29]. To our knowledge, carotid artery intima–media thickness (IMT) and pulse wave velocity (PWV) are two indices of vascular hypertension-mediated organ damage; the former is considered a marker of early atherosclerosis and the latter assesses arterial stiffness [30,31]. Tatasciore et al. revealed that short-term SBP variability correlates significantly with IMT and PWV in hypertensive patients, which implies that increased BPV contributes to the occurrence and development of arterial stiffness and early atherosclerosis [32].

A complicated mechanism might underlie the decreased BPV induced by long-term ECP treatment. It may be related to increased shear stress, improvement in endothelial function, inhibition of oxidative stress and inflammation, and the promotion of vasculogenesis and angiogenesis. Intimal hyperplasia was observed in the coronary arteries of pigs on a high-cholesterol diet, whereas in pigs receiving long-term ECP, the intima-to-media area ratio was significantly decreased by 41.59%. Animal experiments showed that ECP reduces hypercholesterolemia-induced endothelial damage, arrests vascular smooth muscle cell proliferation and migration, decreases the proliferating cell nuclear antigen proliferative index, suppresses extracellular matrix formation, and eventually inhibits intimal hyperplasia and the development of atherosclerosis by increasing the arterial wall shear stress [33]. Kern et al. showed that counterpulation-induced increases in blood flow translate into enhanced endothelial shear stress, which represents a major stimulus for endothelial nitric oxide release and vasodilation [34]. Shear stress also modulates the release of endothelin-1. Longer exposure to higher shear stress levels is associated with a reduction in endothelial endothelin-1 release [35]. Sardina et al. demonstrated that ECP decreases advanced glycation end products (AGE)/receptors for AGE (RAGE) concentrations, inflammation, as measured by hsCRP and soluble vascular cell adhesion molecules-1 (sVCAM-1), and oxidative stress, as measured by 8-iso-prostaglandin 2α (8-iso-PGF2α) in patients with a clinical diagnosis of type II diabetes mellitus [36]. There is increasing evidence that rapid beat-to-beat oscillations of BP are attributable to the interplay of different cardiovascular control systems, including the baroreceptor reflex, the RAS, the vascular myogenic response, and the release of nitric oxide from the endothelium [23]. The effect of decreased beat-to-beat BPV induced by standard ECP treatment is largely attributable to the enhancement of endothelial function and the release of nitric oxide. In addition, cerebral autoregulation ensures the constancy of cerebral blood flow under fluctuating cerebral perfusion pressure, which is impaired after stroke and gradually improves following stroke rehabilitation [37,38]. ECP augments cerebral blood flow in ischemic stroke patients, possibly via impaired cerebral autoregulation [18]. Sykora et al. mentioned that the cross-linked impairment of cerebrovascular autoregulation and altered autonomic drive may be responsible for impaired cerebral perfusion relative to metabolic and inflammatory reactions. Autonomic dysfunction in acute stroke may have effects on outcomes via inadequate cerebral perfusion due to increased BPV and impaired cerebral autoregulation [39]. The effect of ECP on beat-to-beat BPV may be related to gradual improvement of cerebral autoregulation after ischemic stroke.

There are several limitations in this study. First, this is a retrospective study with a small sample size from a single center; thus, the efficacy evidence may be overinterpreted. Second, the use of antihypertensive treatment might confound the BPV analysis, and the proportion of females is really low in both groups. Of note, the drug use and sex were no significantly different between the two groups. However, the gender balance is worth exploring in future studies. Third, the localization of the infarct may influence the BPV. Last, this study mainly investigates the effects of standard ECP treatment on beat-to-beat BPV. Although none of the recruited patients reported in-hospital mortality or early neurological deterioration, the functional outcome results of ECP-treated patients are not included. However, we recently initiated a randomized controlled trial with conventional medical treatment of stroke patients used as controls to investigate the effects of standard ECP treatment on ischemic stroke patients. The functional outcome results from that study will be more convincing. Furthermore, we only investigated two time-point measurements of beat-to-beat BPV, which may not accurately represent the profile of BPV over time following acute ischemic stroke. No longitudinal follow-up at 1, 2, 6, or 12 months was performed to assess the durability of the observed effects or their clinical relevance. As a result, future studies with multicenter, large-sample randomized controlled trials would be an ideal direction. A longitudinal study with multiple time-point measurements and follow-up would be of value.

## 5. Conclusions

A standard 35-session ECP therapy decreases the beat-to-beat BPV without changing SBP and DBP in patients with recent ischemic stroke. ECP may inhibit sympathetic overactivity in terms of the LF/HF ratio of BPV, which reflects a potential impact of ECP treatment on autonomic dysfunction to benefit post-stroke clinical outcomes.

## Figures and Tables

**Table 1:** Baseline characteristics.

	Control Group (n = 20)	ECP Group (n = 20)	*p* Value *
Sex, male/female	18/2	17/3	0.799
Age (mean ± SD), years	62.6 ± 6.6	63.8 ± 11.4	0.820
Hypertension, n (%)	17 (85.0)	18 (90.0)	0.799
DM, n (%)	7 (35.0)	9 (45.0)	0.602
Hyperlipidemia, n (%)	9 (45.0)	8 (40.0)	0.602
Prior stroke, n (%)	7 (35.0)	4 (20.0)	0.429
Smoker, n (%)	11 (55.0)	7 (35.0)	0.289
Drinker, n (%)	6 (30.0)	1 (5.0)	0.183
Admission NIHSS (mean ± SD)	6.3 ± 3.5	6.8 ± 3.7	0.565
Infarct location			1.000
Anterior circulation infarct	15 (75.0)	16 (80.0)	
Posterior circulation infarct	5 (25.0)	4 (20.0)	
Stroke subtypes			0.091
Large artery disease	14 (70.0)	19 (95.0)	
Small vessel disease	6 (30.0)	1 (5.0)	
Antiplatelet, n (%)	20 (100.0)	20 (100.0)	1.000
ACEI, n (%)	4 (20.0)	3 (15.0)	0.799
β-blocker, n (%)	4 (20.0)	5 (25.0)	0.799
Calcium-channel blocker, n (%)	11 (55.0)	5 (25.0)	0.108
Statin, n (%)	20 (100.0)	17 (85.0)	0.429

DM, diabetes mellitus; NIHSS, National Institutes of Health Stroke Scale; ACEI, angiotensin-converting enzyme inhibitor; * Comparison between patients with or without ECP treatment; For *p* values, Pearson χ^2^ test, Fisher exact 2-sided test, and Student *t* test were used when appropriate.

**Table 2:** Blood pressure changes before and after long-term intervention in control and ECP groups.

Parameter	SBP (mmHg)			DBP (mmHg)		
	Control Group	ECP Group	*p*	Control Group	ECP Group	*p*
before intervention	126.30 ± 18.61	134.80 ± 17.25	0.123	81.05 ± 12.46	84.30 ± 15.77	0.659
after intervention	114.61 ± 11.17	120.94 ± 15.41	0.213	76.19 ± 7.70	76.19 ± 11.55	0.779
Delta	11.70 ± 16.18	13.77 ± 19.60	0.116	4.91 ± 11.28	8.09 ± 14.34	0.097
*p*	0.006	0.012		0.073	0.023	

SBP, systolic blood pressure; DBP, diastolic blood pressure; ECP, external counterpulsation.

**Table 3:** Diastolic BPV before and after long-term intervention between control and ECP groups.

	Control Group				ECP Group				*p* *
	Before Intervention	After Intervention	Change (%)	*p*	Before Intervention	After Intervention	Change (%)	*p*	
VLF, mmHg^2^	2.94 (3.35)	0.86 (4.62)	−58.46 (108.52)	0.079	4.58 (8.65)	1.50 (2.08)	−67.86 (57.26)	<0.001	0.110
LF, mmHg^2^	0.85 (2.96)	0.65 (1.40)	−56.11 (50.66)	0.014	3.23 (3.56)	1.25 (1.64)	−51.83 (58.26)	0.005	0.499
HF, mmHg^2^	0.55 (1.06)	0.26 (0.34)	−59.13 (60.76)	0.002	0.84 (1.28)	0.36 (0.32)	−64.22 (34.01)	0.007	0.787
TP, mmHg^2^	4.41 (10.35)	1.73 (5.50)	−50.57 (58.44)	0.008	11.83 (13.13)	3.82 (3.74)	−67.80 (47.90)	0.001	0.130
LF/HF	1.39 (3.55)	4.12 (3.03)	95.13 (301.19)	0.028	3.54 (3.46)	2.87 (2.71)	−13.33 (173.60)	0.433	0.021

VLF, very low frequency; LF, low frequency; HF, high frequency; TP, total power spectral; ECP, external counterpulsation. Data of BPV were shown as median (interquartile range). *p*: comparison between pre- and after intervention; *p* *: comparison in the percentage of BPV changes between medical and ECP groups.

**Table 4:** Systolic BPV before and after long-term intervention between control and ECP groups.

	Control Group				ECP Group				*p* *
	Before Intervention	After Intervention	Change (%)	*p*	Before Intervention	After Intervention	Change (%)	*p*	
VLF, mmHg^2^	5.78 (4.83)	2.95 (6.63)	−16.73 (113.57)	0.411	6.85 (14.51)	3.56 (4.11)	−77.85 (95.29)	0.007	0.051
LF, mmHg^2^	1.79 (6.19)	2.05 (3.14)	−30.56 (124.38)	0.167	5.09 (7.61)	2.03 (2.50)	−51.20 (87.65)	0.025	0.130
HF, mmHg^2^	2.04 (3.46)	0.93 (2.02)	−56.17 (49.04)	0.011	2.26 (2.77)	1.07 (1.80)	−55.17 (68.42)	0.005	0.957
TP, mmHg^2^	10.31 (19.37)	7.77 (8.80)	−11.95 (63.76)	0.100	14.53 (17.23)	6.61 (6.46)	−70.49 (75.22)	0.011	0.048
LF/HF	1.35 (1.52)	2.58 (3.62)	71.82 (266.11)	0.010	2.73 (4.17)	1.73 (2.28)	0.52 (101.43)	0.411	0.004

VLF, very low frequency; LF, low frequency; HF, high frequency; TP, total Power spectral; ECP, external counterpulsation. Data of BPV were shown as median (Interquartile Range). *p*: comparison between pre- and after intervention; *p* *: comparison in the percentage of BPV changes between medical and ECP groups.

## Data Availability

All data generated or analyzed during this study are included in this published article. The raw data that support the findings of this study are available from the corresponding author upon reasonable request.
